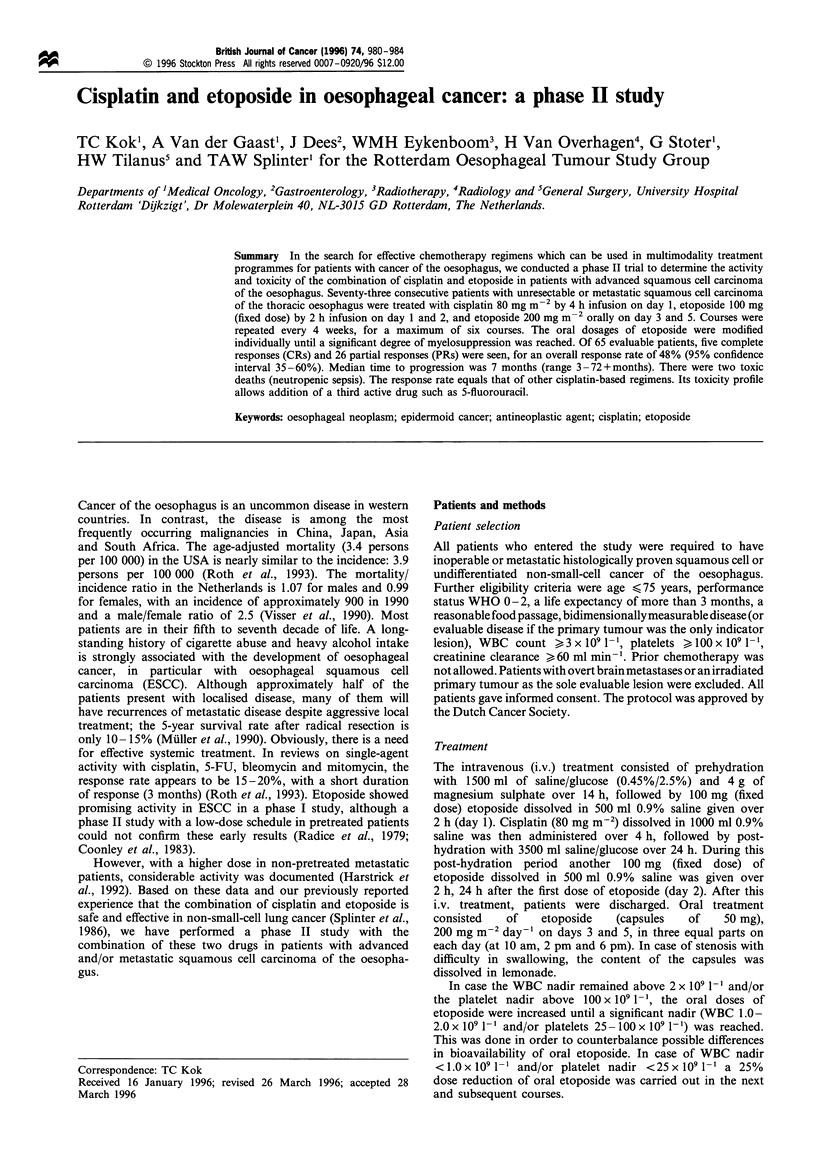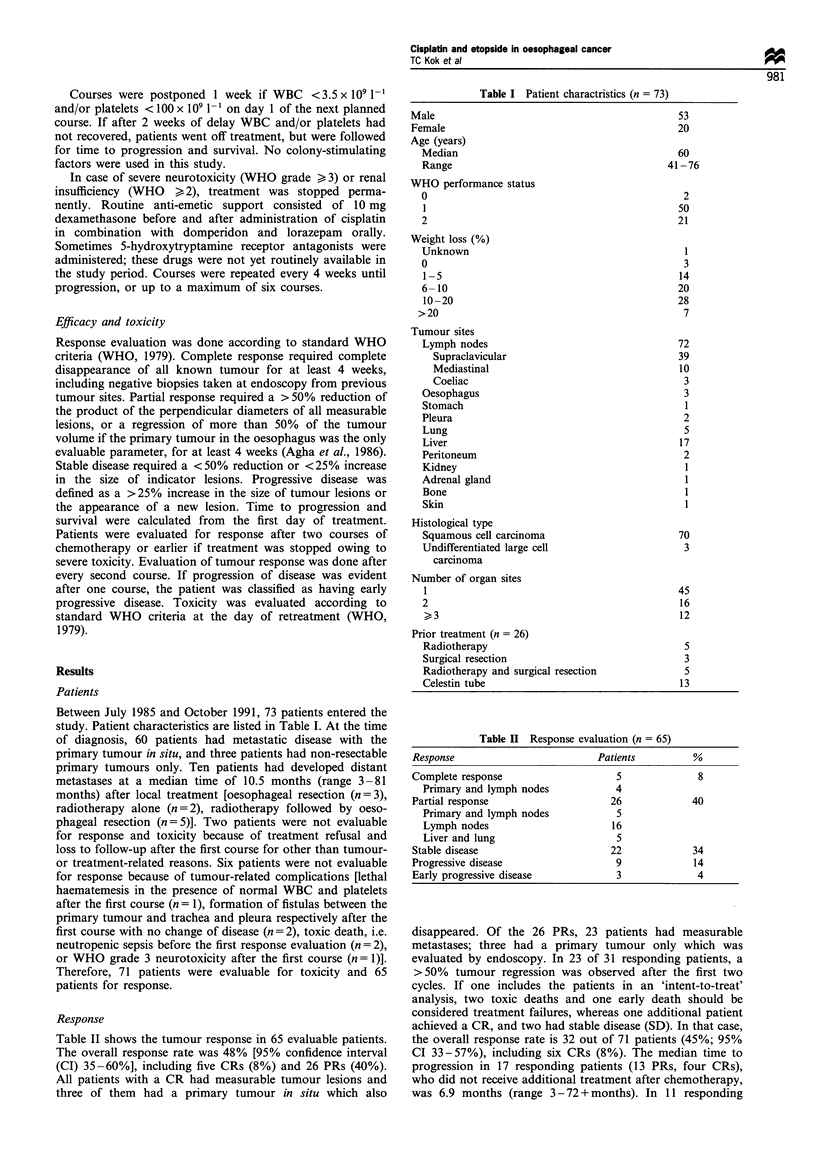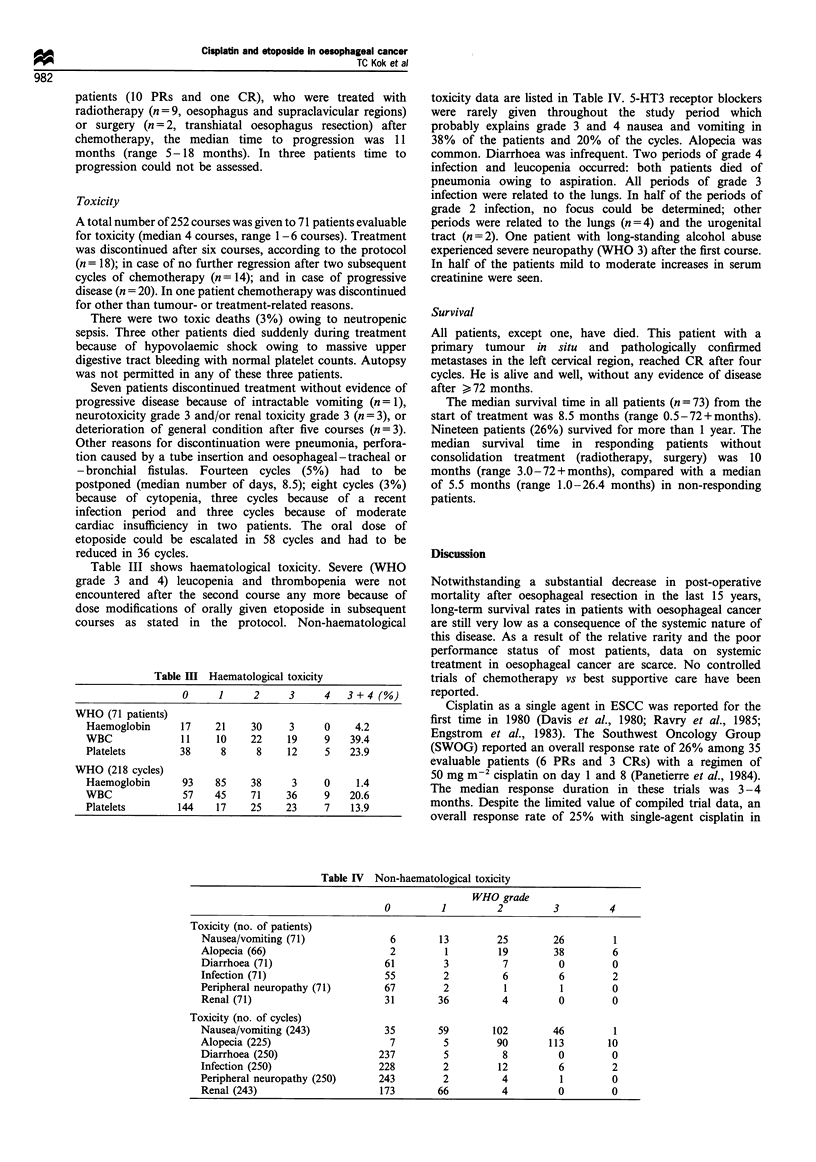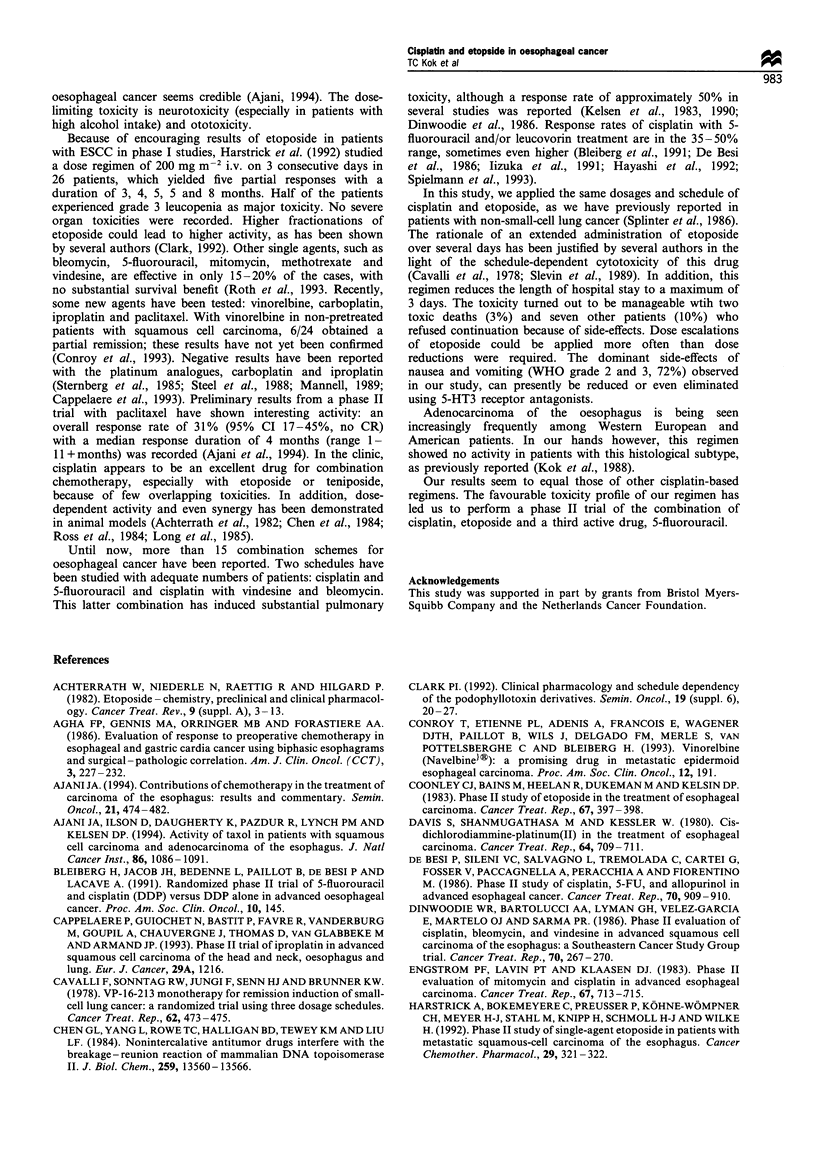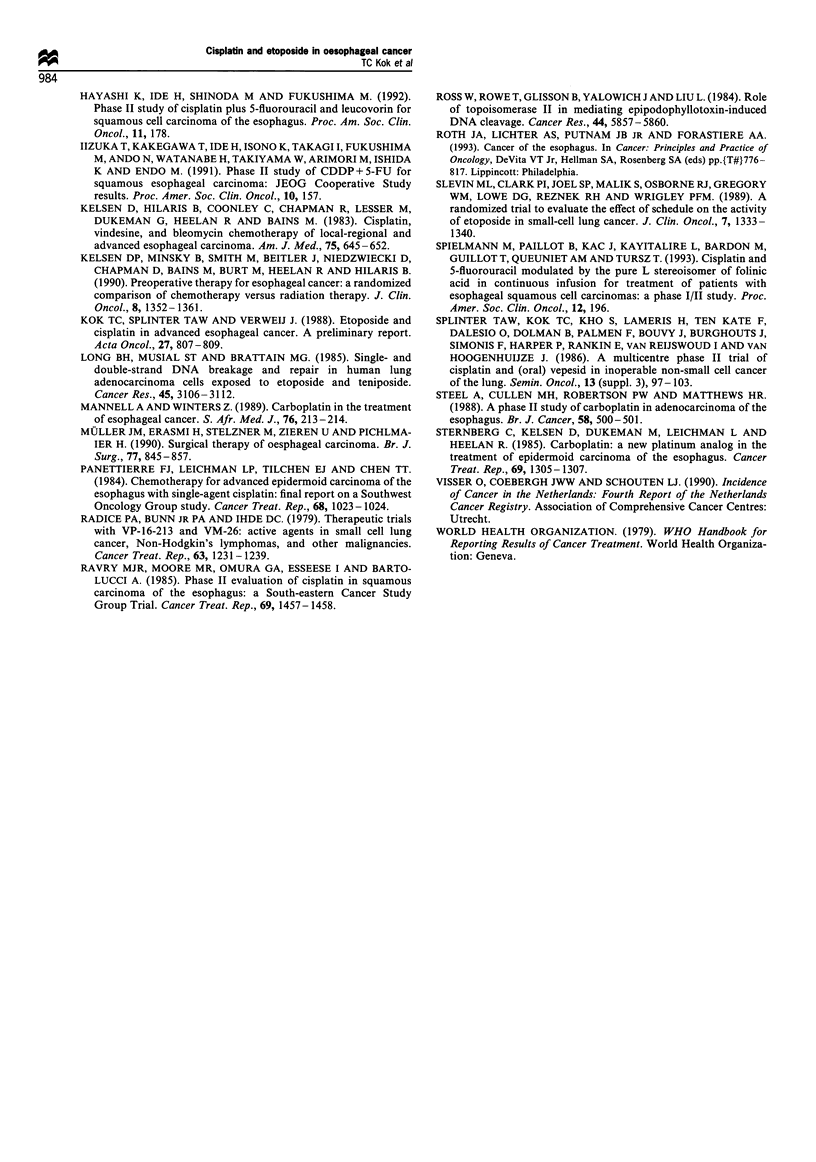# Cisplatin and etoposide in oesophageal cancer: a phase II study. Rotterdam Oesophageal Tumour Study Group.

**DOI:** 10.1038/bjc.1996.469

**Published:** 1996-09

**Authors:** T. C. Kok, A. Van der Gaast, J. Dees, W. M. Eykenboom, H. Van Overhagen, G. Stoter, H. W. Tilanus, T. A. Splinter

**Affiliations:** Department of Medical Oncology, University Hospital Rotterdam, The Netherlands.

## Abstract

In the search for effective chemotherapy regimens which can be used in multimodality treatment programmes for patients with cancer of the oesophagus, we conducted a phase II trial to determine the activity and toxicity of the combination of cisplatin and etoposide in patients with advanced squamous cell carcinoma of the oesophagus. Seventy-three consecutive patients with unresectable or metastatic squamous cell carcinoma of the thoracic oesophagus were treated with cisplatin 80 mg m-2 by 4 h infusion on day 1, etoposide 100 mg (fixed dose) by 2 h infusion on day 1 and 2, and etoposide 200 mg m-2 orally on day 3 and 5. Courses were repeated every 4 weeks, for a maximum of six courses. The oral dosages of etoposide were modified individually until a significant degree of myelosuppression was reached. Of 65 evaluable patients, five complete responses (CRs) and 26 partial responses (PRs) were seen, for an overall response rate of 48% (95% confidence interval 35-60%). Median time to progression was 7 months (range 3-72 + months). There were two toxic deaths (neutropenic sepsis). The response rate equals that of other cisplatin-based regimens. Its toxicity profile allows addition of a third active drug such as 5-fluorouracil.


British Journal of Cancer (1996) 74, 980-984
© 1996 Stockton Press All rights reserved 0007-0920/96 $12.00

### Cisplatin and etoposide in oesophageal cancer: a phase II study

TC Kok[1], A Van der Gaast[1], J Dees[2], WMH Eykenboom[3], H Van Overhagen[4], G Stoter[1], HW Tilanus[5] and TAW Splinter[1] for the Rotterdam Oesophageal Tumour Study Group

*Departments of [1]Medical Oncology, [2]Gastroenterology, [3]Radiotherapy, [4]Radiology and [5]General Surgery, University Hospital Rotterdam 'Dijkzigt', Dr Molewaterplein 40, NL-3015 GD Rotterdam, The Netherlands.*

**Summary** In the search for effective chemotherapy regimens which can be used in multimodality treatment programmes for patients with cancer of the oesophagus, we conducted a phase II trial to determine the activity and toxicity of the combination of cisplatin and etoposide in patients with advanced squamous cell carcinoma of the oesophagus. Seventy-three consecutive patients with unresectable or metastatic squamous cell carcinoma of the thoracic oesophagus were treated with cisplatin 80 mg $m^{-2}$ by 4 h infusion on day 1, etoposide 100 mg (fixed dose) by 2 h infusion on day 1 and 2, and etoposide 200 mg $m^{-2}$ orally on day 3 and 5. Courses were repeated every 4 weeks, for a maximum of six courses. The oral dosages of etoposide were modified individually until a significant degree of myelosuppression was reached. Of 65 evaluable patients, five complete responses (CRs) and 26 partial responses (PRs) were seen, for an overall response rate of 48% (95% confidence interval 35-60%). Median time to progression was 7 months (range 3-72+months). There were two toxic deaths (neutropenic sepsis). The response rate equals that of other cisplatin-based regimens. Its toxicity profile allows addition of a third active drug such as 5-fluorouracil.

**Keywords:** oesophageal neoplasm; epidermoid cancer; antineoplastic agent; cisplatin; etoposide

Cancer of the oesophagus is an uncommon disease in western countries. In contrast, the disease is among the most frequently occurring malignancies in China, Japan, Asia and South Africa. The age-adjusted mortality (3.4 persons per 100 000) in the USA is nearly similar to the incidence: 3.9 persons per 100 000 (Roth *et al.*, 1993). The mortality/incidence ratio in the Netherlands is 1.07 for males and 0.99 for females, with an incidence of approximately 900 in 1990 and a male/female ratio of 2.5 (Visser *et al.*, 1990). Most patients are in their fifth to seventh decade of life. A long-standing history of cigarette abuse and heavy alcohol intake is strongly associated with the development of oesophageal cancer, in particular with oesophageal squamous cell carcinoma (ESCC). Although approximately half of the patients present with localised disease, many of them will have recurrences of metastatic disease despite aggressive local treatment; the 5-year survival rate after radical resection is only 10-15% (Müller *et al.*, 1990). Obviously, there is a need for effective systemic treatment. In reviews on single-agent activity with cisplatin, 5-FU, bleomycin and mitomycin, the response rate appears to be 15-20%, with a short duration of response (3 months) (Roth *et al.*, 1993). Etoposide showed promising activity in ESCC in a phase I study, although a phase II study with a low-dose schedule in pretreated patients could not confirm these early results (Radice *et al.*, 1979; Coonley *et al.*, 1983).

However, with a higher dose in non-pretreated metastatic patients, considerable activity was documented (Harstrick *et al.*, 1992). Based on these data and our previously reported experience that the combination of cisplatin and etoposide is safe and effective in non-small-cell lung cancer (Splinter *et al.*, 1986), we have performed a phase II study with the combination of these two drugs in patients with advanced and/or metastatic squamous cell carcinoma of the oesophagus.

Correspondence: TC Kok
Received 16 January 1996; revised 26 March 1996; accepted 28 March 1996

### Patients and methods

### *Patient selection*

All patients who entered the study were required to have inoperable or metastatic histologically proven squamous cell or undifferentiated non-small-cell cancer of the oesophagus. Further eligibility criteria were age ⩽75 years, performance status WHO 0-2, a life expectancy of more than 3 months, a reasonable food passage, bidimensionally measurable disease (or evaluable disease if the primary tumour was the only indicator lesion), WBC count $\geqslant 3\times10^9$ $l^{-1}$, platelets $\geqslant 100\times10^9$ $l^{-1}$, creatinine clearance ⩾60 ml $min^{-1}$. Prior chemotherapy was not allowed. Patients with overt brain metastases or an irradiated primary tumour as the sole evaluable lesion were excluded. All patients gave informed consent. The protocol was approved by the Dutch Cancer Society.

### *Treatment*

The intravenous (i.v.) treatment consisted of prehydration with 1500 ml of saline/glucose (0.45%/2.5%) and 4 g of magnesium sulphate over 14 h, followed by 100 mg (fixed dose) etoposide dissolved in 500 ml 0.9% saline given over 2 h (day 1). Cisplatin (80 mg $m^{-2}$) dissolved in 1000 ml 0.9% saline was then administered over 4 h, followed by post-hydration with 3500 ml saline/glucose over 24 h. During this post-hydration period another 100 mg (fixed dose) of etoposide dissolved in 500 ml 0.9% saline was given over 2 h, 24 h after the first dose of etoposide (day 2). After this i.v. treatment, patients were discharged. Oral treatment consisted of etoposide (capsules of 50 mg), 200 mg $m^{-2}$ $day^{-1}$ on days 3 and 5, in three equal parts on each day (at 10 am, 2 pm and 6 pm). In case of stenosis with difficulty in swallowing, the content of the capsules was dissolved in lemonade.

In case the WBC nadir remained above $2\times10^9$ $l^{-1}$ and/or the platelet nadir above $100\times10^9$ $l^{-1}$, the oral doses of etoposide were increased until a significant nadir (WBC 1.0-$2.0\times10^9$ $l^{-1}$ and/or platelets $25-100\times10^9$ $l^{-1}$) was reached. This was done in order to counterbalance possible differences in bioavailability of oral etoposide. In case of WBC nadir $<1.0\times10^9$ $l^{-1}$ and/or platelet nadir $<25\times10^9$ $l^{-1}$ a 25% dose reduction of oral etoposide was carried out in the next and subsequent courses.

Courses were postponed 1 week if WBC $<3.5 \times 10^9 l^{-1}$ and/or platelets $<100 \times 10^9 l^{-1}$ on day 1 of the next planned course. If after 2 weeks of delay WBC and/or platelets had not recovered, patients went off treatment, but were followed for time to progression and survival. No colony-stimulating factors were used in this study.

In case of severe neurotoxicity (WHO grade $\geqslant 3$) or renal insufficiency (WHO $\geqslant 2$), treatment was stopped permanently. Routine anti-emetic support consisted of 10 mg dexamethasone before and after administration of cisplatin in combination with domperidon and lorazepam orally. Sometimes 5-hydroxytryptamine receptor antagonists were administered; these drugs were not yet routinely available in the study period. Courses were repeated every 4 weeks until progression, or up to a maximum of six courses.

### *Efficacy and toxicity*

Response evaluation was done according to standard WHO criteria (WHO, 1979). Complete response required complete disappearance of all known tumour for at least 4 weeks, including negative biopsies taken at endoscopy from previous tumour sites. Partial response required a >50% reduction of the product of the perpendicular diameters of all measurable lesions, or a regression of more than 50% of the tumour volume if the primary tumour in the oesophagus was the only evaluable parameter, for at least 4 weeks (Agha *et al.*, 1986). Stable disease required a <50% reduction or <25% increase in the size of indicator lesions. Progressive disease was defined as a >25% increase in the size of tumour lesions or the appearance of a new lesion. Time to progression and survival were calculated from the first day of treatment. Patients were evaluated for response after two courses of chemotherapy or earlier if treatment was stopped owing to severe toxicity. Evaluation of tumour response was done after every second course. If progression of disease was evident after one course, the patient was classified as having early progressive disease. Toxicity was evaluated according to standard WHO criteria at the day of retreatment (WHO, 1979).

### Results

### *Patients*

Between July 1985 and October 1991, 73 patients entered the study. Patient characteristics are listed in Table I. At the time of diagnosis, 60 patients had metastatic disease with the primary tumour *in situ*, and three patients had non-resectable primary tumours only. Ten patients had developed distant metastases at a median time of 10.5 months (range 3–81 months) after local treatment [oesophageal resection ($n=3$), radiotherapy alone ($n=2$), radiotherapy followed by oesophageal resection ($n=5$)]. Two patients were not evaluable for response and toxicity because of treatment refusal and loss to follow-up after the first course for other than tumour- or treatment-related reasons. Six patients were not evaluable for response because of tumour-related complications [lethal haematemesis in the presence of normal WBC and platelets after the first course ($n=1$), formation of fistulas between the primary tumour and trachea and pleura respectively after the first course with no change of disease ($n=2$), toxic death, i.e. neutropenic sepsis before the first response evaluation ($n=2$), or WHO grade 3 neurotoxicity after the first course ($n=1$)]. Therefore, 71 patients were evaluable for toxicity and 65 patients for response.

### *Response*

Table II shows the tumour response in 65 evaluable patients. The overall response rate was 48% [95% confidence interval (CI) 35–60%], including five CRs (8%) and 26 PRs (40%). All patients with a CR had measurable tumour lesions and three of them had a primary tumour *in situ* which also disappeared. Of the 26 PRs, 23 patients had measurable metastases; three had a primary tumour only which was evaluated by endoscopy. In 23 of 31 responding patients, a >50% tumour regression was observed after the first two cycles. If one includes the patients in an 'intent-to-treat' analysis, two toxic deaths and one early death should be considered treatment failures, whereas one additional patient achieved a CR, and two had stable disease (SD). In that case, the overall response rate is 32 out of 71 patients (45%; 95% CI 33–57%), including six CRs (8%). The median time to progression in 17 responding patients (13 PRs, four CRs), who did not receive additional treatment after chemotherapy, was 6.9 months (range 3–72+months). In 11 responding

**Table I** Patient charactristics ($n$ = 73)

| | |
|---|---|
| Male | 53 |
| Female | 20 |
| Age (years) | |
| Median | 60 |
| Range | 41–76 |
| WHO performance status | |
| 0 | 2 |
| 1 | 50 |
| 2 | 21 |
| Weight loss (%) | |
| Unknown | 1 |
| 0 | 3 |
| 1–5 | 14 |
| 6–10 | 20 |
| 10–20 | 28 |
| >20 | 7 |
| Tumour sites | |
| Lymph nodes | 72 |
| Supraclavicular | 39 |
| Mediastinal | 10 |
| Coeliac | 3 |
| Oesophagus | 3 |
| Stomach | 1 |
| Pleura | 2 |
| Lung | 5 |
| Liver | 17 |
| Peritoneum | 2 |
| Kidney | 1 |
| Adrenal gland | 1 |
| Bone | 1 |
| Skin | 1 |
| Histological type | |
| Squamous cell carcinoma | 70 |
| Undifferentiated large cell carcinoma | 3 |
| Number of organ sites | |
| 1 | 45 |
| 2 | 16 |
| $\geqslant 3$ | 12 |
| Prior treatment ($n$ = 26) | |
| Radiotherapy | 5 |
| Surgical resection | 3 |
| Radiotherapy and surgical resection | 5 |
| Celestin tube | 13 |

**Table II** Response evaluation ($n$ = 65)

| *Response* | *Patients* | *%* |
|---|---|---|
| Complete response | 5 | 8 |
| Primary and lymph nodes | 4 | |
| Partial response | 26 | 40 |
| Primary and lymph nodes | 5 | |
| Lymph nodes | 16 | |
| Liver and lung | 5 | |
| Stable disease | 22 | 34 |
| Progressive disease | 9 | 14 |
| Early progressive disease | 3 | 4 |

patients (10 PRs and one CR), who were treated with radiotherapy ($n=9$, oesophagus and supraclavicular regions) or surgery ($n=2$, transhiatal oesophagus resection) after chemotherapy, the median time to progression was 11 months (range 5–18 months). In three patients time to progression could not be assessed.

### *Toxicity*

A total number of 252 courses was given to 71 patients evaluable for toxicity (median 4 courses, range 1–6 courses). Treatment was discontinued after six courses, according to the protocol ($n=18$); in case of no further regression after two subsequent cycles of chemotherapy ($n=14$); and in case of progressive disease ($n=20$). In one patient chemotherapy was discontinued for other than tumour- or treatment-related reasons.

There were two toxic deaths (3%) owing to neutropenic sepsis. Three other patients died suddenly during treatment because of hypovolaemic shock owing to massive upper digestive tract bleeding with normal platelet counts. Autopsy was not permitted in any of these three patients.

Seven patients discontinued treatment without evidence of progressive disease because of intractable vomiting ($n=1$), neurotoxicity grade 3 and/or renal toxicity grade 3 ($n=3$), or deterioration of general condition after five courses ($n=3$). Other reasons for discontinuation were pneumonia, perforation caused by a tube insertion and oesophageal–tracheal or –bronchial fistulas. Fourteen cycles (5%) had to be postponed (median number of days, 8.5); eight cycles (3%) because of cytopenia, three cycles because of a recent infection period and three cycles because of moderate cardiac insufficiency in two patients. The oral dose of etoposide could be escalated in 58 cycles and had to be reduced in 36 cycles.

Table III shows haematological toxicity. Severe (WHO grade 3 and 4) leucopenia and thrombopenia were not encountered after the second course any more because of dose modifications of orally given etoposide in subsequent courses as stated in the protocol. Non-haematological toxicity data are listed in Table IV. 5-HT3 receptor blockers were rarely given throughout the study period which probably explains grade 3 and 4 nausea and vomiting in 38% of the patients and 20% of the cycles. Alopecia was common. Diarrhoea was infrequent. Two periods of grade 4 infection and leucopenia occurred: both patients died of pneumonia owing to aspiration. All periods of grade 3 infection were related to the lungs. In half of the periods of grade 2 infection, no focus could be determined; other periods were related to the lungs ($n=4$) and the urogenital tract ($n=2$). One patient with long-standing alcohol abuse experienced severe neuropathy (WHO 3) after the first course. In half of the patients mild to moderate increases in serum creatinine were seen.

**Table III** Haematological toxicity

| | *0* | *1* | *2* | *3* | *4* | *3 + 4 (%)* |
|---|---|---|---|---|---|---|
| WHO (71 patients) | | | | | | |
| Haemoglobin | 17 | 21 | 30 | 3 | 0 | 4.2 |
| WBC | 11 | 10 | 22 | 19 | 9 | 39.4 |
| Platelets | 38 | 8 | 8 | 12 | 5 | 23.9 |
| WHO (218 cycles) | | | | | | |
| Haemoglobin | 93 | 85 | 38 | 3 | 0 | 1.4 |
| WBC | 57 | 45 | 71 | 36 | 9 | 20.6 |
| Platelets | 144 | 17 | 25 | 23 | 7 | 13.9 |

### *Survival*

All patients, except one, have died. This patient with a primary tumour *in situ* and pathologically confirmed metastases in the left cervical region, reached CR after four cycles. He is alive and well, without any evidence of disease after ⩾72 months.

The median survival time in all patients ($n=73$) from the start of treatment was 8.5 months (range 0.5–72+months). Nineteen patients (26%) survived for more than 1 year. The median survival time in responding patients without consolidation treatment (radiotherapy, surgery) was 10 months (range 3.0–72+months), compared with a median of 5.5 months (range 1.0–26.4 months) in non-responding patients.

### Discussion

Notwithstanding a substantial decrease in post-operative mortality after oesophageal resection in the last 15 years, long-term survival rates in patients with oesophageal cancer are still very low as a consequence of the systemic nature of this disease. As a result of the relative rarity and the poor performance status of most patients, data on systemic treatment in oesophageal cancer are scarce. No controlled trials of chemotherapy *vs* best supportive care have been reported.

Cisplatin as a single agent in ESCC was reported for the first time in 1980 (Davis *et al.*, 1980; Ravry *et al.*, 1985; Engstrom *et al.*, 1983). The Southwest Oncology Group (SWOG) reported an overall response rate of 26% among 35 evaluable patients (6 PRs and 3 CRs) with a regimen of 50 mg $m^{-2}$ cisplatin on day 1 and 8 (Panetierre *et al.*, 1984). The median response duration in these trials was 3–4 months. Despite the limited value of compiled trial data, an overall response rate of 25% with single-agent cisplatin in

**Table IV** Non-haematological toxicity

| | | | *WHO grade* | | |
|---|---|---|---|---|---|
| | *0* | *1* | *2* | *3* | *4* |
| Toxicity (no. of patients) | | | | | |
| Nausea/vomiting (71) | 6 | 13 | 25 | 26 | 1 |
| Alopecia (66) | 2 | 1 | 19 | 38 | 6 |
| Diarrhoea (71) | 61 | 3 | 7 | 0 | 0 |
| Infection (71) | 55 | 2 | 6 | 6 | 2 |
| Peripheral neuropathy (71) | 67 | 2 | 1 | 1 | 0 |
| Renal (71) | 31 | 36 | 4 | 0 | 0 |
| Toxicity (no. of cycles) | | | | | |
| Nausea/vomiting (243) | 35 | 59 | 102 | 46 | 1 |
| Alopecia (225) | 7 | 5 | 90 | 113 | 10 |
| Diarrhoea (250) | 237 | 5 | 8 | 0 | 0 |
| Infection (250) | 228 | 2 | 12 | 6 | 2 |
| Peripheral neuropathy (250) | 243 | 2 | 4 | 1 | 0 |
| Renal (243) | 173 | 66 | 4 | 0 | 0 |

oesophageal cancer seems credible (Ajani, 1994). The dose-limiting toxicity is neurotoxicity (especially in patients with high alcohol intake) and ototoxicity.

Because of encouraging results of etoposide in patients with ESCC in phase I studies, Harstrick *et al.* (1992) studied a dose regimen of 200 mg $m^{-2}$ i.v. on 3 consecutive days in 26 patients, which yielded five partial responses with a duration of 3, 4, 5, 5 and 8 months. Half of the patients experienced grade 3 leucopenia as major toxicity. No severe organ toxicities were recorded. Higher fractionations of etoposide could lead to higher activity, as has been shown by several authors (Clark, 1992). Other single agents, such as bleomycin, 5-fluorouracil, mitomycin, methotrexate and vindesine, are effective in only 15–20% of the cases, with no substantial survival benefit (Roth *et al.*, 1993. Recently, some new agents have been tested: vinorelbine, carboplatin, iproplatin and paclitaxel. With vinorelbine in non-pretreated patients with squamous cell carcinoma, 6/24 obtained a partial remission; these results have not yet been confirmed (Conroy *et al.*, 1993). Negative results have been reported with the platinum analogues, carboplatin and iproplatin (Sternberg *et al.*, 1985; Steel *et al.*, 1988; Mannell, 1989; Cappelaere *et al.*, 1993). Preliminary results from a phase II trial with paclitaxel have shown interesting activity: an overall response rate of 31% (95% CI 17–45%, no CR) with a median response duration of 4 months (range 1–11+months) was recorded (Ajani *et al.*, 1994). In the clinic, cisplatin appears to be an excellent drug for combination chemotherapy, especially with etoposide or teniposide, because of few overlapping toxicities. In addition, dose-dependent activity and even synergy has been demonstrated in animal models (Achterrath *et al.*, 1982; Chen *et al.*, 1984; Ross *et al.*, 1984; Long *et al.*, 1985).

Until now, more than 15 combination schemes for oesophageal cancer have been reported. Two schedules have been studied with adequate numbers of patients: cisplatin and 5-fluorouracil and cisplatin with vindesine and bleomycin. This latter combination has induced substantial pulmonary toxicity, although a response rate of approximately 50% in several studies was reported (Kelsen *et al.*, 1983, 1990; Dinwoodie *et al.*, 1986. Response rates of cisplatin with 5-fluorouracil and/or leucovorin treatment are in the 35–50% range, sometimes even higher (Bleiberg *et al.*, 1991; De Besi *et al.*, 1986; Iizuka *et al.*, 1991; Hayashi *et al.*, 1992; Spielmann *et al.*, 1993).

In this study, we applied the same dosages and schedule of cisplatin and etoposide, as we have previously reported in patients with non-small-cell lung cancer (Splinter *et al.*, 1986). The rationale of an extended administration of etoposide over several days has been justified by several authors in the light of the schedule-dependent cytotoxicity of this drug (Cavalli *et al.*, 1978; Slevin *et al.*, 1989). In addition, this regimen reduces the length of hospital stay to a maximum of 3 days. The toxicity turned out to be manageable wtih two toxic deaths (3%) and seven other patients (10%) who refused continuation because of side-effects. Dose escalations of etoposide could be applied more often than dose reductions were required. The dominant side-effects of nausea and vomiting (WHO grade 2 and 3, 72%) observed in our study, can presently be reduced or even eliminated using 5-HT3 receptor antagonists.

Adenocarcinoma of the oesophagus is being seen increasingly frequently among Western European and American patients. In our hands however, this regimen showed no activity in patients with this histological subtype, as previously reported (Kok *et al.*, 1988).

Our results seem to equal those of other cisplatin-based regimens. The favourable toxicity profile of our regimen has led us to perform a phase II trial of the combination of cisplatin, etoposide and a third active drug, 5-fluorouracil.

**Acknowledgements**
This study was supported in part by grants from Bristol Myers-Squibb Company and the Netherlands Cancer Foundation.

### References

ACHTERRATH W, NIEDERLE N, RAETTIG R AND HILGARD P. (1982). Etoposide – chemistry, preclinical and clinical pharmacology. *Cancer Treat. Rev.*, **9** (suppl. A), 3–13.

AGHA FP, GENNIS MA, ORRINGER MB AND FORASTIERE AA. (1986). Evaluation of response to preoperative chemotherapy in esophageal and gastric cardia cancer using biphasic esophagrams and surgical–pathologic correlation. *Am. J. Clin. Oncol. (CCT)*, **3**, 227–232.

AJANI JA. (1994). Contributions of chemotherapy in the treatment of carcinoma of the esophagus: results and commentary. *Semin. Oncol.*, **21**, 474–482.

AJANI JA, ILSON D, DAUGHERTY K, PAZDUR R, LYNCH PM AND KELSEN DP. (1994). Activity of taxol in patients with squamous cell carcinoma and adenocarcinoma of the esophagus. *J. Natl Cancer Inst.*, **86**, 1086–1091.

BLEIBERG H, JACOB JH, BEDENNE L, PAILLOT B, DE BESI P AND LACAVE A. (1991). Randomized phase II trial of 5-fluorouracil and cisplatin (DDP) versus DDP alone in advanced oesophageal cancer. *Proc. Am. Soc. Clin. Oncol.*, **10**, 145.

CAPPELAERE P, GUIOCHET N, BASTIT P, FAVRE R, VANDERBURG M, GOUPIL A, CHAUVERGNE J, THOMAS D, VAN GLABBEKE M AND ARMAND JP. (1993). Phase II trial of iproplatin in advanced squamous cell carcinoma of the head and neck, oesophagus and lung. *Eur. J. Cancer*, **29A**, 1216.

CAVALLI F, SONNTAG RW, JUNGI F, SENN HJ AND BRUNNER KW. (1978). VP-16-213 monotherapy for remission induction of small-cell lung cancer: a randomized trial using three dosage schedules. *Cancer Treat. Rep.*, **62**, 473–475.

CHEN GL, YANG L, ROWE TC, HALLIGAN BD, TEWEY KM AND LIU LF. (1984). Nonintercalative antitumor drugs interfere with the breakage–reunion reaction of mammalian DNA topoisomerase II. *J. Biol. Chem.*, **259**, 13560–13566.

CLARK PI. (1992). Clinical pharmacology and schedule dependency of the podophyllotoxin derivatives. *Semin. Oncol.*, **19** (suppl. 6), 20–27.

CONROY T, ETIENNE PL, ADENIS A, FRANCOIS E, WAGENER DJTH, PAILLOT B, WILS J, DELGADO FM, MERLE S, VAN POTTELSBERGHE C AND BLEIBERG H. (1993). Vinorelbine (Navelbine[J]®): a promising drug in metastatic epidermoid esophageal carcinoma. *Proc. Am. Soc. Clin. Oncol.*, **12**, 191.

COONLEY CJ, BAINS M, HEELAN R, DUKEMAN M AND KELSIN DP. (1983). Phase II study of etoposide in the treatment of esophageal carcinoma. *Cancer Treat. Rep.*, **67**, 397–398.

DAVIS S, SHANMUGATHASA M AND KESSLER W. (1980). Cis-dichlorodiammine-platinum(II) in the treatment of esophageal carcinoma. *Cancer Treat. Rep.*, **64**, 709–711.

DE BESI P, SILENI VC, SALVAGNO L, TREMOLADA C, CARTEI G, FOSSER V, PACCAGNELLA A, PERACCHIA A AND FIORENTINO M. (1986). Phase II study of cisplatin, 5-FU, and allopurinol in advanced esophageal cancer. *Cancer Treat. Rep.*, **70**, 909–910.

DINWOODIE WR, BARTOLUCCI AA, LYMAN GH, VELEZ-GARCIA E, MARTELO OJ AND SARMA PR. (1986). Phase II evaluation of cisplatin, bleomycin, and vindesine in advanced squamous cell carcinoma of the esophagus: a Southeastern Cancer Study Group trial. *Cancer Treat. Rep.*, **70**, 267–270.

ENGSTROM PF, LAVIN PT AND KLAASEN DJ. (1983). Phase II evaluation of mitomycin and cisplatin in advanced esophageal carcinoma. *Cancer Treat. Rep.*, **67**, 713–715.

HARSTRICK A, BOKEMEYERE C, PREUSSER P, KÖHNE-WÖMPNER CH, MEYER H-J, STAHL M, KNIPP H, SCHMOLL H-J AND WILKE H. (1992). Phase II study of single-agent etoposide in patients with metastatic squamous-cell carcinoma of the esophagus. *Cancer Chemother. Pharmacol.*, **29**, 321–322.

HAYASHI K, IDE H, SHINODA M AND FUKUSHIMA M. (1992). Phase II study of cisplatin plus 5-fluorouracil and leucovorin for squamous cell carcinoma of the esophagus. *Proc. Am. Soc. Clin. Oncol.*, **11**, 178.

IIZUKA T, KAKEGAWA T, IDE H, ISONO K, TAKAGI I, FUKUSHIMA M, ANDO N, WATANABE H, TAKIYAMA W, ARIMORI M, ISHIDA K AND ENDO M. (1991). Phase II study of CDDP + 5-FU for squamous esophageal carcinoma: JEOG Cooperative Study results. *Proc. Amer. Soc. Clin. Oncol.*, **10**, 157.

KELSEN D, HILARIS B, COONLEY C, CHAPMAN R, LESSER M, DUKEMAN G, HEELAN R AND BAINS M. (1983). Cisplatin, vindesine, and bleomycin chemotherapy of local-regional and advanced esophageal carcinoma. *Am. J. Med.*, **75**, 645–652.

KELSEN DP, MINSKY B, SMITH M, BEITLER J, NIEDZWIECKI D, CHAPMAN D, BAINS M, BURT M, HEELAN R AND HILARIS B. (1990). Preoperative therapy for esophageal cancer: a randomized comparison of chemotherapy versus radiation therapy. *J. Clin. Oncol.*, **8**, 1352–1361.

KOK TC, SPLINTER TAW AND VERWEIJ J. (1988). Etoposide and cisplatin in advanced esophageal cancer. A preliminary report. *Acta Oncol.*, **27**, 807–809.

LONG BH, MUSIAL ST AND BRATTAIN MG. (1985). Single- and double-strand DNA breakage and repair in human lung adenocarcinoma cells exposed to etoposide and teniposide. *Cancer Res.*, **45**, 3106–3112.

MANNELL A AND WINTERS Z. (1989). Carboplatin in the treatment of esophageal cancer. *S. Afr. Med. J.*, **76**, 213–214.

MÜLLER JM, ERASMI H, STELZNER M, ZIEREN U AND PICHLMAIER H. (1990). Surgical therapy of oesphageal carcinoma. *Br. J. Surg.*, **77**, 845–857.

PANETTIERRE FJ, LEICHMAN LP, TILCHEN EJ AND CHEN TT. (1984). Chemotherapy for advanced epidermoid carcinoma of the esophagus with single-agent cisplatin: final report on a Southwest Oncology Group study. *Cancer Treat. Rep.*, **68**, 1023–1024.

RADICE PA, BUNN JR PA AND IHDE DC. (1979). Therapeutic trials with VP-16-213 and VM-26: active agents in small cell lung cancer, Non-Hodgkin's lymphomas, and other malignancies. *Cancer Treat. Rep.*, **63**, 1231–1239.

RAVRY MJR, MOORE MR, OMURA GA, ESSEESE I AND BARTOLUCCI A. (1985). Phase II evaluation of cisplatin in squamous carcinoma of the esophagus: a South-eastern Cancer Study Group Trial. *Cancer Treat. Rep.*, **69**, 1457–1458.

ROSS W, ROWE T, GLISSON B, YALOWICH J AND LIU L. (1984). Role of topoisomerase II in mediating epipodophyllotoxin-induced DNA cleavage. *Cancer Res.*, **44**, 5857–5860.

ROTH JA, LICHTER AS, PUTNAM JB JR AND FORASTIERE AA. (1993). Cancer of the esophagus. In *Cancer: Principles and Practice of Oncology*, DeVita VT Jr, Hellman SA, Rosenberg SA (eds) pp.{T#}776–817. Lippincott: Philadelphia.

SLEVIN ML, CLARK PI, JOEL SP, MALIK S, OSBORNE RJ, GREGORY WM, LOWE DG, REZNEK RH AND WRIGLEY PFM. (1989). A randomized trial to evaluate the effect of schedule on the activity of etoposide in small-cell lung cancer. *J. Clin. Oncol.*, **7**, 1333–1340.

SPIELMANN M, PAILLOT B, KAC J, KAYITALIRE L, BARDON M, GUILLOT T, QUEUNIET AM AND TURSZ T. (1993). Cisplatin and 5-fluorouracil modulated by the pure L stereoisomer of folinic acid in continuous infusion for treatment of patients with esophageal squamous cell carcinomas: a phase I/II study. *Proc. Amer. Soc. Clin. Oncol.*, **12**, 196.

SPLINTER TAW, KOK TC, KHO S, LAMERIS H, TEN KATE F, DALESIO O, DOLMAN B, PALMEN F, BOUVY J, BURGHOUTS J, SIMONIS F, HARPER P, RANKIN E, VAN REIJSWOUD I AND VAN HOOGENHUIJZE J. (1986). A multicentre phase II trial of cisplatin and (oral) vepesid in inoperable non-small cell cancer of the lung. *Semin. Oncol.*, **13** (suppl. 3), 97–103.

STEEL A, CULLEN MH, ROBERTSON PW AND MATTHEWS HR. (1988). A phase II study of carboplatin in adenocarcinoma of the esophagus. *Br. J. Cancer*, **58**, 500–501.

STERNBERG C, KELSEN D, DUKEMAN M, LEICHMAN L AND HEELAN R. (1985). Carboplatin: a new platinum analog in the treatment of epidermoid carcinoma of the esophagus. *Cancer Treat. Rep.*, **69**, 1305–1307.

VISSER O, COEBERGH JWW AND SCHOUTEN LJ. (1990). *Incidence of Cancer in the Netherlands: Fourth Report of the Netherlands Cancer Registry*. Association of Comprehensive Cancer Centres: Utrecht.

WORLD HEALTH ORGANIZATION. (1979). *WHO Handbook for Reporting Results of Cancer Treatment*. World Health Organization: Geneva.